# Parkin-Dependent Mitophagy Is Required for the Inhibition of ATF4 on NLRP3 Inflammasome Activation in Cerebral Ischemia-Reperfusion Injury in Rats

**DOI:** 10.3390/cells8080897

**Published:** 2019-08-14

**Authors:** Qi He, Zhenyu Li, Changchang Meng, Jingxian Wu, Yong Zhao, Jing Zhao

**Affiliations:** 1 Department of Pathophysiology, Chongqing Medical University, Chongqing 400010, China; 2 Institute of Neuroscience, Chongqing Medical University, Chongqing 400010, China; 3 Department of Pathology, Chongqing Medical University, Chongqing 400010, China

**Keywords:** ATF4, Parkin, mitophagy, NLRP3 inflammasome, ischemia-reperfusion, neuroprotection

## Abstract

**Background**: Nod-like receptor protein 3 (NLRP3) inflammasome is a crucial contributor in the inflammatory process during cerebral ischemia/reperfusion (I/R) injury. ATF4 plays a pivotal role in the pathogenesis of cerebral I/R injury, however, its function and underlying mechanism are not fully characterized yet. In the current study, we examined whether ATF4 ameliorates cerebral I/R injury by inhibiting NLRP3 inflammasome activation and whether mitophagy is involved in this process. In addition, we explored the role of parkin in ATF4-mediated protective effects. **Method**: To address these issues, healthy male adult Sprague-Dawley rats were exposed to middle cerebral artery occlusion for 1 h followed by 24 h reperfusion. Adeno-associated virus (AAV) and siRNA were injected into rats to overexpress and knockdown ATF4 expression, respectively. After pretreatment with AAV, mdivi-1(mitochondrial division inhibitor-1) was injected into rats to block mitophagy activity. Parkin expression was knockdown using specific siRNA after AAV pretreatment. **Result**: Data showed that ATF4 overexpression induced by AAV was protective against cerebral I/R injury, as evidenced by reduced cerebral infraction volume, decreased neurological scores and improved outcomes of HE and Nissl staining. In addition, overexpression of ATF4 gene was able to up-regulate Parkin expression, enhance mitophagy activity and inhibit NLRP3 inflammasome-mediated inflammatory response. ATF4 knockdown induced by siRNA resulted in the opposite effects. Furthermore, ATF4-mediated inhibition of NLRP3 inflammasome activation was strongly affected by mitophagy blockage upon mdivi-1 injection. Besides, ATF4-mediated increase of mitophagy activity and inhibition of NLRP3 inflammasome activation were effectively reversed by Parkin knockdown using siRNA. **Conclusion**: Our study demonstrated that ATF4 is able to alleviate cerebral I/R injury by suppressing NLRP3 inflammasome activation through parkin-dependent mitophagy activity. These results may provide a new strategy to relieve cerebral I/R injury by modulating mitophagy-NLRP3 inflammasome axis.

## 1. Introduction

Cerebral ischemia-reperfusion (I/R) injury is a severe health problem worldwide. Although the mechanism of cerebral I/R injury is complex [1,2,3], inflammatory damage has a critical impact on the pathogenesis of cerebral I/R injury and may become a potentially therapeutic target [4,5,6]. NLRP3 is an important tissue-damage sensor and it was reported to be crucial in triggering sterile inflammation. Among several agonists of the NLRP3 inflammasome, damaged mitochondria and their released mitochondrial signals, such as mitochondrial DNA (mtDNA) and mitochondrial reactive oxygen species (mtROS), contribute to NLRP3 inflammasome activation [7,8,9]. Upon induction, NLRP3 directly binds to the adaptor molecule ASC, which subsequently recruits and activates pro-caspase-1. This interaction leads to the formation of a cytoplasmic multiprotein complex, termed NLRP3 inflammasome, which is able to cleave the pro-IL-1β and pro-IL-18 into their active forms, cleaved IL-1β and cleaved IL-18, and so to initiate and amplify the inflammatory response [1,10]. Dysfunction in NLRP3 inflammasome activity is known to correlate with several diseases [11,12,13,14], such as Alzheimer’s disease, type II diabetes mellitus, atherosclerosis, and cancer. Importantly, recent studies demonstrated that NLRP3 inflammasome activation can contribute to the pathological development of cerebral I/R injury [15,16].

Mitophagy is an important mechanism to guarantee mitochondrial quality. During mitophagy, dysfunctional mitochondria can be specifically recognized, targeted and degraded through the autophagy process [17]. Accumulating evidence reported that a defective mitophagy is linked to the pathogenesis of neurodegenerative diseases, inflammation, and other metabolic disorders [18,19,20,21,22]. Furthermore, it has been recently observed that mitophagy may exert a neuroprotective effect during cerebral ischemia-reperfusion injury [3] and it can negatively regulate NLRP3 inflammasome activation [23]. Therefore, we want to assess whether mitophagy induction could alleviate cerebral ischemia-reperfusion injury by eliminating dysfunctional mitochondria and inhibiting NLRP3 inflammasome activation.

PINK1/Parkin partnership is a well-described regulator of mitophagy. It has been recently observed that parkin can be recruited to the mitochondria through the interaction with PINK1 protein upon mitochondrial depolarization. Parkin can ubiquitinate several mitochondrial outer membrane proteins through its E3 ligase activity in order to recruit p62 protein, which interacts with LC3, leading to the accomplishment of mitophagy [24]. Noteworthy, it has been reported that ATF4, a transcription factor involved in ER stress, can up-regulate parkin expression [25,26,27]. Moreover, previous evidence has also indicated that ATF4 can promote mitophagy activity by promoting parkin expression [28].

For this reason, we therefore want to assess whether ATF4 could protect against cerebral I/R injury by affecting NLRP3 inflammasome activation via the parkin-mitophagy axis.

## 2. Materials and Methods

### 2.1. Animals

Healthy male adult Sprague–Dawley rats weighing 250–300 g were supplied by the Center of Experimental Animal in Chongqing Medical University, Chongqing, China. All animals used in this experiment were cared for in strict accordance with the Guide for the Care and Use of Laboratory Animals (NIH Publication No. 85-23, revised 1996). All animal experimental procedures were approved by the Ethics Committee of Experimental Animals of Chongqing Medical University. All measures were made to minimize animal suffering.

### 2.2. Reagents and Antibodies

Mdivi-1(mitochondrial division inhibitor-1) and TTC (2,3,5-triphenyltetrazolium chloride) were purchased from Sigma Aldrich (St. Louis, Missouri, MO, USA); BSA (bovine serum protein) was obtained from BOSTER (Boster Biological Technology, Wuhan, China, AR1006). Blots were incubated with antibodies against ATF4 (Proteintech, Rosemont, Pennsylvania, USA, 10835-1-AP), Parkin (CST, Boston, Massachusetts, USA, #4211), TOM20 (Proteintech, USA, 11802-1-AP), COX4I1 (Proteintech, USA, 60251-1-Ig), NLRP3 inflammasome (Affinity, Cincinnati, Ohio, USA, DF7438), pro-caspase-1 (ABclonal, Wuhan, China, A0964), cleaved caspase-1 (CST, USA, #67314), pro-IL-1β (ABclonal, China, A11370), cleaved IL-1β (Affinity, USA, AF4006), pro-IL-18 (Proteintech, USA, 10663-1-AP), cleaved IL-18 (R&D Systems, Minneapolis, Minnesota, USA, AF521) and β-actin (ABclonal, China, AC004), followed by the secondary antibodies conjugated to horseradish peroxidase anti-rabbit IgG (H + L) (ABclonal, China, AS014) and anti-mouse IgG (H + L) (ABclonal, China, AS003).

The primary antibodies used for the immunofluorescence analysis were against TOM20 (Proteintech, USA, 11802-1-AP) and COX4I1 proteins (Proteintech, USA, 60251-1-Ig). Brain sections were then incubated with the secondary antibodies goat anti-rabbit DyLight 488 (Abbkine, green, A23240) and goat anti-mouse DyLight 549 (Abbkine, red, A23310).

### 2.3. Stereotaxic Injection of AAV Vectors

The AAV vectors for ATF4 overexpression were designed and produced by OBiO Technology Corporation, Shanghai, China. The procedure of AAV vectors stereotaxic injection was chosen according to a previous study [29,30]. Briefly, 5-week-old rats were intraperitoneally injected with 4% chloral hydrate (400 mg/kg) and then restrained in a stereotaxic apparatus (RWD life Science, Shenzhen, China). AAV Vectors encoding ATF4 (5.0 × 10^12^ v.g./mL) and empty AAV vectors encoding GFP were injected into the left cortex at nine sites ( 2 uL per site, 0.4 μL/min ) by a micro-injection pump. During this procedure, a homeothermic heating blanket was used to maintain body temperature. After injection, the needle was kept in site for 5 min and then slowly withdrawn. Five weeks later, animals underwent middle cerebral artery occlusion (MCAO) surgery.

The stereotaxic coordinates selected for multiple sites injections of AAV vectors were as follows: site 1, 0.3 mm anterior to bregma, 3 mm lateral to midline, 2 mm beneath the surface of the skull; site 2, 0.3 mm anterior to bregma, 5 mm lateral to midline, 3 mm beneath the surface of the skull; site 3, 0.3 mm anterior to bregma, 5 mm lateral to midline, 6 mm beneath the surface of the skull; site 4, 0.8 mm posterior to bregma, 3 mm lateral to midline, 2 mm beneath the surface of the skull; site 5, 0.8 mm posterior to bregma, 5 mm lateral to midline, 3 mm beneath the surface of the skull; site 6, 0.8 mm posterior to bregma, 5 mm lateral to midline, 6 mm beneath the surface of the skull; site 7, 1.9 mm posterior to bregma, 3 mm lateral to midline, 2 mm beneath the surface of the skull; site 8, 1.9 mm posterior to bregma, 5 mm lateral to midline, 3 mm beneath the surface of the skull; site 9, 1.9 mm posterior to bregma, 5 mm lateral to midline, 6 mm beneath the surface of the skull.

### 2.4. Intracerebroventricular Injection of siRNA and Mdivi-1 Administration

ATF4 siRNA (sense primer 5-GCUGCUUAUAUUACUCUAATT-3 and antisense primer 5-UUAGAGUAAUAUAAGCAGCTT-3) and parkin siRNA (sense primer 5-CCAACUCCCUGAUUAAAGATT-3 and antisense primer 5-UCUUUAAUCAGGGAGUUGGTT-3) were designed and chemically synthesized by GenePharma Corporation, Shanghai, China. The RNase-free water was used to dissolve siRNA reaching the final concentration of 2 μg/µL. The intracerebroventricular injection of siRNA was performed as previously described [31]. Briefly, rats were placed in a stereotaxic apparatus (RWD life Science, Shenzhen, China) after being anesthetized with 4% chloral hydrate (400 mg/kg, i.p) and the bregma point was then exposed. A Hamilton syringe of 25 µL volume was perpendicularly inserted at 1.0 mm posterior to bregma, 2.0 mm lateral to midline, and 3.5 mm below the skull surface. Afterwards, 15 μL of a solution containing ATF4 siRNA or parkin siRNA (2 μg/μL, 1 μL/min) was injected into the left lateral ventricle. The needle was kept in place for 10 min and then withdrawn slowly. MCAO model was established after 24 h upon siRNA injection.

Mdivi-1 (mitochondrial division inhibitor-1) administration (10 mg/kg, i.p.) was performed at the onset of reperfusion as previously described [3].

### 2.5. MCAO Model in Rats

Transient cerebral ischemia-reperfusion was obtained through the MCAO procedure [31]. In brief, the experimental rats were anesthetized with chloral hydrate (4%, 400 mg/kg, i.p.) and subsequently placed on a heating pad to keep body temperature. The left common and external carotid arteries were then proximally exposed and ligated after a ventral midline neck incision. A 5-0 nylon monofilament (Beijing Cinontech Co.Ltd. Beijing, China) with a blunt-end coated with poly-l-lysine was inserted into the internal carotid artery and forwarded into the middle cerebral artery origin until meeting a slight resistance. Meanwhile, the regional cerebral blood flow (CBF) was decreased to <20% of the baseline level. Reperfusion was achieved after 1 hour of occlusion by slowly withdrawing the nylon monofilament and the blood flow was recovered to 75% of baseline. Sham animals received the same operation without MCAO. All animals were sacrificed after 24 h upon reperfusion.

### 2.6. Cerebral Infarct Volume Evaluation

Animals were anesthetized with 4% chloral hydrate (400 mg/kg, i.p) at 24 h after reperfusion and sacrificed. The whole brain was then rapidly removed, frozen for 15 min at −20 °C in an adult rat brain matrix (RWD life Science, Shenzhen, China) and then cut into five coronal sections (2 mm thick). Cryosections were stained with 2% TTC for 15 min at room temperature in the dark, followed by fixation with 4% paraformaldehyde at 4 °C for 48 h. Red signal indicates normal tissue, while white signal indicates infarct tissue. Slides were acquired via a digital camera and analyzed by ImageJ analysis software (version 6.0, NIH, Bethesda, National Institutes of Health, Maryland, USA). The percentage of corrected infarct volume was calculated as follows: {[total lesion volume − (ipsilateral hemisphere volume − contralateral hemisphere volume)]/contralateral hemisphere volume} × 100.

### 2.7. Assessment of Neurological Score

The neurological damage was determined in blinded fashion on the basis of neurological deficits as previously described [31]: Briefly, animals receiving a score of 0 show normal behavior; a score of 1 indicates the inability to fully extend the contralateral forelimb and torso turning to the contralateral side when held by tail; a score of 2 refers to animals which circle to the contralateral side, but exhibit normal posture at rest; a score of 3 indicates animals which lean to the injured side; a score of 4 refers to animals with no spontaneous locomotor activity and a depressed level of consciousness; while a score of 5 identifies dead animals. The experimental rats receiving scores of 0 or 5 were excluded from the analysis.

### 2.8. Western Blotting

After saline perfusion, the cortices of brains were rapidly removed and homogenized in RIPA buffer containing phenylmethylsulfonyl fluoride (PMSF, 1mM/mL). Lysates were then centrifuged at 15,000× *g* for 8 min at 4 °C, and the supernatants were subsequently collected and stored at −80 °C. Equal amounts of protein lysates (30 µg) were separated on sodium dodecyl sulphate (SDS)-polyacrylamide gel electrophoresis and then electrotransferred to PVDF membranes (Millipore, Boston, Massachusetts, USA). Membranes were blocked with 5% non-fat milk at room temperature for 90 min and incubated with primary antibodies at 4 °C overnight. The HRP-conjugated secondary antibodies were then applied to the membranes for 1 h at room temperature.

### 2.9. Immunofluorescence and Confocal Microscopy

Brains were rapidly removed from animals after saline perfusion, fixed in 4% paraformaldehyde for 24 h at 4 °C, dehydrated in a 30% sucrose solution at 4 °C for 48 h, and then frozen at −80 °C. Brains were then cut into frozen sections (10 µm thick) using the freezing microtome (Leica, CM1850, Heidelberg, Germany), blocked with blocking buffer (3% BSA + 1% Triton X-100 in PBS) for 1 h at room temperature and then incubated with primary antibodies diluted in blocking buffer against TOM20 and COX4I1 at 4 °C overnight. After being washed thrice with PBS, the sections were incubated with secondary antibodies labeled with Dylight at 37 °C for 60 min. Finally, the coverslips were mounted on VECTASHIELD Hardset mounting medium with DAPI (Vector lab, Burlingame, California, USA, H-1500). Immunofluorescence images were analyzed and acquired on a confocal microscope (Nikon A1*R, Tokyo, Japan).

### 2.10. Electron Microscopy

Brains were quickly removed from experimental animals after 2.5% glutaric dialdehyde perfusion. A small sample of cortices were isolated from the area of infarction and then fixed in 2.5% glutaric dialdehyde. The analysis of the ultrastructure of the mitochondria and autophagosome surrounding the mitochondria was performed by the Life Sciences Academy of Chongqing medical university. Images were acquired by transmission electron microscopy (JEM-1400PLUS, Tokyo, Japan).

### 2.11. HE Staining and Nissl Staining

Brains were removed, fixed and dehydrated as described above. Samples were embedded in paraffin and then cut into sections. The HE staining and Nissl staining were performed according to the operating manual.

### 2.12. Determination of Total Reactive Oxygen Species (ROS) in Tissue Levels 

The Detection Kit of Reactive Oxygen Species in Tissue was obtained from BestBio (BestBio Science, shanghai, China, BB470532), and the experimental procedures were performed according to the manufacture’s instruction. In brief, equal amounts of fresh brain cortices (50 mg) were washed with PBS and then homogenized in 1 mL lysis buffer-A using the glass homogenizer. Next, the homogenates were centrifuged at 1000× *g* for 10 min at 4 °C and the supernatants were collected. The BBcellprobeTMDDO1 (10 µL), a new fluorescent probe containing 2,7-Dichlorodihydrofluorescein diacetate (DCFH-DA), was then added to the supernatants (190 µL), and samples were incubated for 30 min at 37 °C in the dark. Finally, green fluorescence intensity was quantified by the automatic fluorescence microplate reader with excitation wavelength 488 nm and emission wavelength at 530 nm. The levels of reactive oxygen species in tissue are represented as the ratio of the fluorescence intensity to the protein concentration.

### 2.13. ELISA of IL-1β and IL-18 Activity

The levels of IL-1β and IL-18 cytokines in the cerebral cortex were detected using specific ELISA kits (Boster Biological Technology, China, EK0592 and EK0393) according to the manufacturer’s instructions. The protein concentration of each sample was used to normalize the level of cytokine in the tissue homogenate after performing the ELISA.

### 2.14. Statistical Analysis

Data are shown as mean ± SD. GraphPad Prism software (version 6.0) was used to complete all statistical analyses. Parametric data were analyzed using one-way analysis of variance (ANOVA) followed by Tukey’s multiple comparisons test. Significance was defined as *p*-values < 0.05.

## 3. Results

### 3.1. ATF4 Protects Against Cerebral Ischemia-Reperfusion Injury in Rats

**Figure 1:**
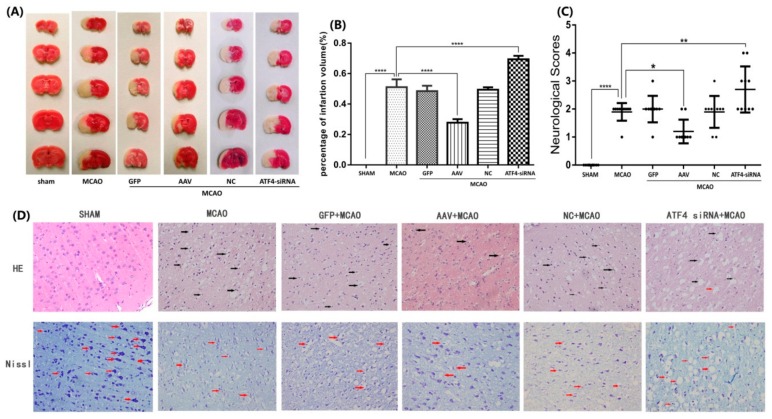
ATF4 prevents cerebral ischemia reperfusion injury in rats. (**A,B**) Infarct volume resulted to be significantly shrunk or enlarged upon AAV or ATF4 siRNA treatment compared to MCAO group. (**C**) Neurological scores was decreased or increased by AAV or ATF4 siRNA compared to MCAO group. (**D**) Representative HE staining and Nissl staining (× 400) of AAV and ATF4 siRNA could effectively improve or aggravate tissue and neurons injury in the cortex compared to MCAO group. The black arrows in HE staining marked the disorderly cytoplasmic loose, edema and karyopyknosis of the neurocytes; the red arrows in Nissl staining marked differently changed nissl bodies or degenerated neurons. Error bars represent mean ± SD. (* *p* < 0.05, ** *p* < 0.01 and **** *p*< 0.0001 vs. indicated group). Panel A, D *n* = 5 in each group; Panel C *n* = 10 in each group. MCAO, middle cerebral artery occlusion-reperfusion; GFP, green fluorescent protein; AAV, ATF4 overexpression adeno-associated virus; siRNA, small interfering RNA; NC, negative control siRNA.

In order to over-express and knockdown ATF4 expression, AAV and siRNA were injected before rats were subjected to middle cerebral artery occlusion-reperfusion (MCAO), respectively. To investigate the potential protection of ATF4 against cerebral ischemia-reperfusion injury, the impact of ATF4 on brain infarct volume and neurological score was examined (Figure 1A–C). The AAV + MCAO group showed a significant reduction of both brain infarct volume and neurological score when compared with the MCAO group. As expected, the ATF4-siRNA + MCAO group, but not the GFP + MCAO group or the NC + MCAO group, showed increased brain infarct volume and neurological score when compared with the MCAO group.

To further confirm the neuroprotective effect of ATF4, sections were stained with HE and Nissl to evaluate morphological alterations. As shown in Figure 1D, the HE staining evidenced a disorderly cytoplasmic loose, edema and karyopyknosis of the neurocytes in the MCAO group as compared with the sham group. Of note, such alterations could be markedly reversed following treatment with AAV. In contrast, ATF4-siRNA treatment resulted in a more serious distributed vacuolization and edema in the interstitial spaces in comparison with the MCAO group. Nissl staining presented decreased and shallow nissl bodies in the MCAO group whereas no changes in the neuron morphology were observed in the sham group. Treatment of rats with AAV injection significantly improved the number and shade of the nissl bodies; oppositely, the ATF4-siRNA group presented a marked increase in the number of degenerated neurons and even neurons loss. However, no significant differences in HE and Nissl staining were detected between the GFP + MCAO group or the NC + MCAO group and the MCAO group.

Altogether, the above results conclusively show that ATF4 may exert a neuroprotective effect against cerebral ischemia-reperfusion injury in rats.

### 3.2. ATF4 Restrains NLRP3 Inflammasome-Mediated Inflammatory Response during Cerebral I/R Injury in Rats

**Figure 2:**
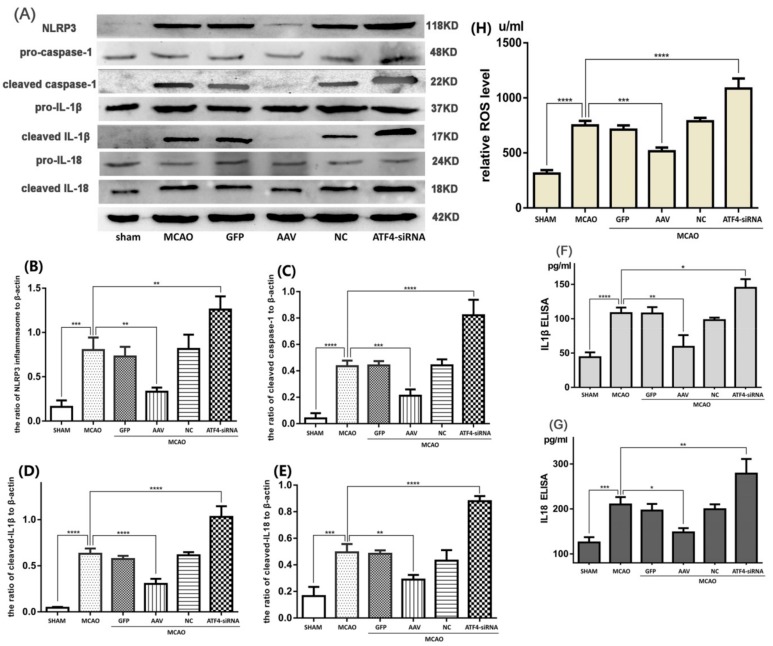
ATF4 affects NLRP3 inflammasome-mediated inflammatory response during cerebral I/R injury. (**A**–**E**) Representative Western blots showing protein levels of NLRP3 protein, cleaved-caspase-1, cleaved-IL-1β and cleaved-IL-18. Protein levels were normalized to β-actin. (**F**,**G**) The cytokine levels of IL-1β and IL-18 were quantified by ELISA assays. (**H**) The levels of ROS production in the cortex were quantified by a commercial Detection Kit of Reactive Oxygen Species in Tissue. Error bars represent mean ± SD. (* *p* < 0.05, ** *p* < 0.01, *** *p* < 0.001 and **** *p*< 0.0001 vs. indicated group). *n* = 5 in each group. MCAO, middle cerebral artery occlusion-reperfusion; GFP, green fluorescent protein; AAV, ATF4 overexpression adeno-associated virus; siRNA, small interfering RNA; NC, negative control siRNA.

As inflammatory response plays a major role in the cerebral I/R injury, we speculated that the neuroprotective effect of ATF4 may be attributable to its role in the inhibition of the NLRP3 inflammasome activation. To test this hypothesis, protein expression of components of activated NLRP3 inflammasome (i.e., cleaved-caspase-1, cleaved-IL-1β and cleaved-IL-18) was assessed by Western blotting. As shown in Figure 2A–E, the MCAO challenge up-regulated the expressions of NLRP3 protein, cleaved-caspase-1, cleaved-IL-1β, and cleaved- IL-18 compared with the sham group. Such an increase was significantly inhibited by treatment with AAV. In contrast, ATF4-siRNA treatment further increased the expressions of activated NLRP3 inflammasome-related components as compared with the MCAO group. Furthermore, to further determine the levels of IL-1β and IL-18, ELISA assays were performed. Data showed a similar tendency with the expressions of cleaved-IL-1β and cleaved-IL-18 (Figure 2F,G). Moreover, the amount of key NLRP3 inflammasome activator (i.e., ROS) was evaluated by a commercial Detection Kit of Reactive Oxygen Species in Tissue (Figure 2H). These results showed that the AAV treatment clearly decreased the production of ROS compared with the MCAO group, whereas treatment with ATF4-siRNA had the opposite effect.

In conclusion, such results imply that ATF4 may ameliorate I/R-induced brain injury by suppressing inflammatory pathways driven by the NLRP3 inflammasome.

### 3.3. ATF4 Increases Parkin Expression and Mitophagy Activity

**Figure 3:**
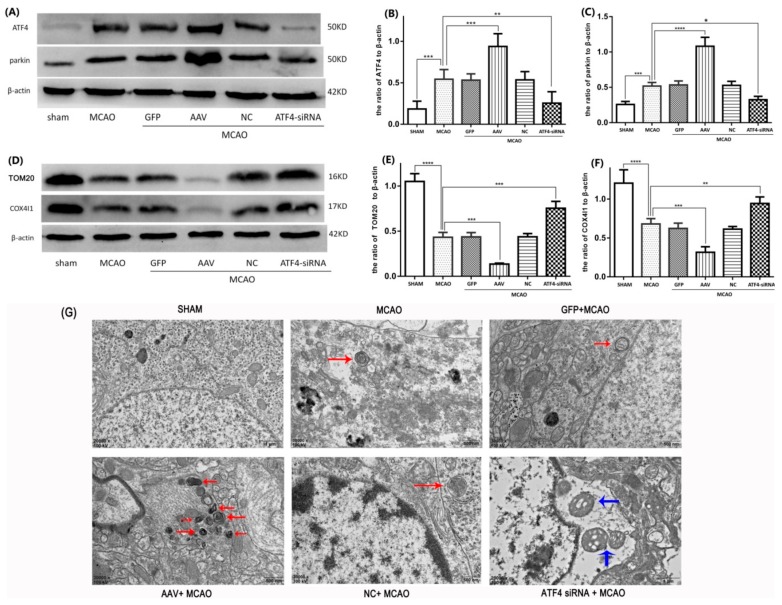
ATF4 increases parkin expression and mitophagy activity during cerebral I/R injury in rats. (**A**–**C**) Representative Western blots of ATF4 and parkin. (**D**–**F**) Representative Western blots of TOM20 and COX4I1. Protein levels were normalized to β-actin. (**G**) The representative ultrastructures of mitophagy are shown: the structures of mitochondrials and nucleus were normal in the sham group; a few mitochondria engulfed by a double-membrane structure are labeled by red arrows in the MCAO, GFP + MCAO and NC + MCAO groups; many autophagic vesicles, which encapsulated with mitochondrias and were fused with lysosomal, were labeled by red arrows in the AAV + MCAO group; in the ATF4-siRNA + MCAO group, swollen mitochondrias were labeled by blue arrows and the typical autophagic vesicle was hardly observed. Error bars represent mean ± SD. (* *p* < 0.05, ** *p* < 0.01, *** *p* < 0.001 and **** *p*< 0.0001 vs. indicated group). *n* = 5 in each group. MCAO, middle cerebral artery occlusion-reperfusion; GFP, green fluorescent protein; AAV, ATF4 overexpression adeno-associated virus; siRNA, small interfering RNA; NC, negative control siRNA.

In our experiments, we used AAV (ATF4 overexpression adeno-associated virus) and ATF4-siRNA (the knockdown efficiency of three different siRNAs on ATF4 expression can be seen in Supplementary Information) to increase and decrease the level of ATF4 expression, respectively. As shown in Figure 3A–C, I/R stimulation boosted ATF4 and parkin protein levels compared to the sham group. When compared with the MCAO group, AAV administration remarkably enhanced ATF4 and parkin expressions, whereas ATF4-siRNA treatment distinctly reduced them. Neither GFP or NC treatment had a significant impact on the levels of ATF4 and parkin expression in comparison to the MCAO group. These data suggest that ATF4 may have the ability to induce parkin expression during cerebral I/R stimulation.

To investigate the effect of ATF4 on mitophagy, the morphology of neurocytes in the cortex was evaluated by electron microscopy. As shown in Figure 3G, in the sham group, both mitochondrial and nuclear structures are normal and double-membrane autophagosomes were not detected. In the MCAO, GFP+MCAO and NC + MCAO groups, only a few mitochondria engulfed by a double-membrane structure, representing the existence of mitophagy, could be observed. Importantly, when treated with AAV, a large number of autophagic vesicles, which were fused with a lysosome, were encapsulated with mitochondrias, indicating a stronger mitophagy activity. In contrast, the ATF4-siRNA treatment not only made it difficult to detect double-membrane autophagosomes encapsulated with mitochondria but also resulted in many swollen mitochondria and small chromatin clots dispersed in the nucleus, which represented a more serious neuron injury.

**Figure 4:**
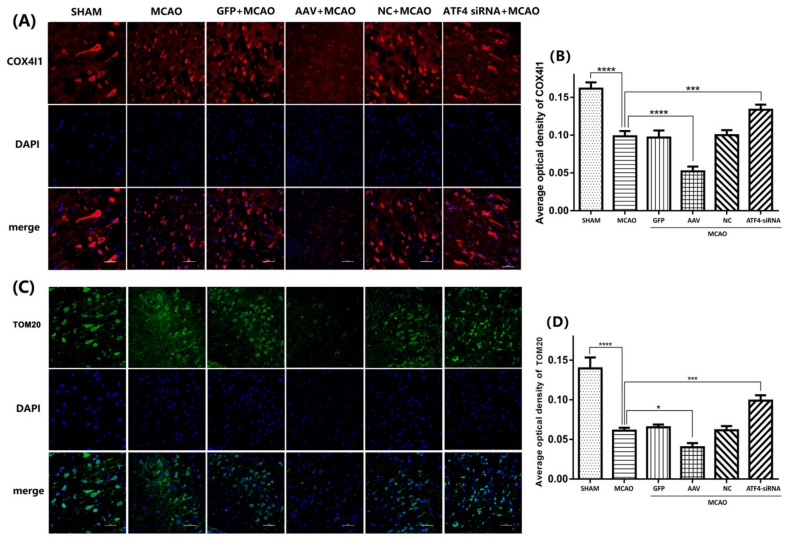
ATF4 decreases TOM20 and COX4I1 expressions in the cortex. (**A**–**D**) TOM20 and COX4I1 immunostaining resulted to be significantly reduced in the MCAO group compared to the sham group. The average optical densities of TOM20 and COX4I1 immunostaining resulted to be decreased or increased by AAV and ATF4 siRNA compared to the MCAO group. Error bars represent mean ± SD. (* *p* < 0.05, *** *p* < 0.001 and **** *p*< 0.0001 vs. indicated group). *n* = 5 in each group. MCAO, middle cerebral artery occlusion-reperfusion; GFP, green fluorescent protein; AAV, ATF4 overexpression adeno-associated virus; siRNA, small interfering RNA; NC, negative control siRNA.

To further confirm these findings, the expressions of two independent mitochondrial markers (i.e., TOM20 and COX4I1) were assessed by Western blotting and Immunostaining. Consistent with our previous findings, a reduced expression of TOM20 and COX4I1 was seen in the MCAO group compared to the sham group and such a decrease was further enhanced by AAV administration. In contrast, ATF4-siRNA treatment largely increased TOM20 and COX4I1 levels compared with the MCAO group (Figure 3D–F). Similar results were obtained by Immunostaining analysis (Figure 4A–D).

Overall, such data indicate that ATF4 is able to increase parkin expression and augment mitophagy activity during cerebral I/R injury.

### 3.4. The ATF4-Mediated Regulation of Mitophagy Relies on Parkin Modulation

**Figure 5:**
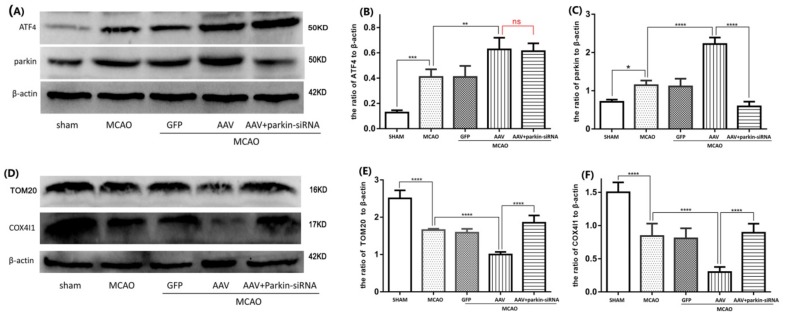
Parkin siRNA significantly impaired the effects of ATF4 on parkin expression and mitophagy activity. Compared with the AAV+MCAO group, parkin siRNA remarkably decreased parkin expression (**A**,**C**), however, it has no obvious influence on ATF4 expression (**A**,**B**). Parkin siRNA apparently increased the levels of TOM20 and COX4I1 compared to the AAV+MCAO group (**D**–**F**). Error bars represent mean ± SD. (* *p* < 0.05, ** *p* < 0.01, *** *p* < 0.001 and **** *p*< 0.0001 vs. indicated group). *n* = 5 in each group. MCAO, middle cerebral artery occlusion-reperfusion; GFP, green fluorescent protein; AAV, ATF4 overexpression adeno-associated virus; siRNA, small interfering RNA.

A crucial role in mitophagy induction is exerted by parkin, whose expression has been shown to be up-regulated by ATF4. To unravel the role of parkin in the ATF4-mediated mitophagy, AAV-injected rats were exposed to I/R stimulus and then treated with parkin-siRNA. As shown in Figure 5A,C, parkin expression in the AAV + MCAO group was remarkably increased compared to that in the MCAO group. Parkin-siRNA treatment significantly reduced the expression of parkin compared to the AAV + MCAO group (the knockdown efficiency of three different siRNAs on parkin expression can be seen in Supplementary Information). This result indicates that parkin-siRNA treatment effectively abrogates the ATF4-driven parkin induction. Furthermore, AAV treatment prominently decreased TOM20 and COX4I1 expression compared with the MCAO group. However, such a decrease was abolished by a subsequent parkin-siRNA treatment (Figure 5D–F), thus indicating that parkin knockdown abolished the ATF4-mediated increase of mitophagy activity. Of note, no significant difference in the level of ATF4 between the AAV+MCAO group and the parkin-siRNA + AAV + MCAO group was observed (Figure 5A,B), thus ruling out a role for Parkin-siRNA treatment in regulating ATF4 expression.

Altogether, these results suggest that ATF4 may enhance mitophagy activity by increasing parkin expression in cerebral I/R injury.

### 3.5. Crucial Role of Parkin in the ATF4-Mediated Inhibition of NLRP3 Inflammasome Activation

**Figure 6:**
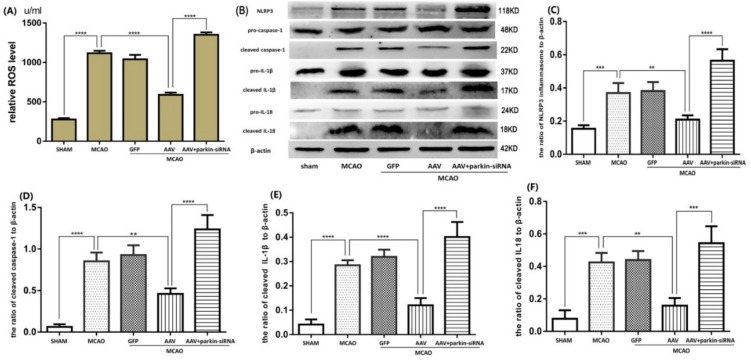
Parkin siRNA reverses the inhibitory effect of ATF4 on NLRP3 inflammasome activation. Representative Western blots showing the levels of NLRP3 protein, cleaved-caspase-1, cleaved-IL-1β and cleaved-IL-18 (**B**–**F**), and histograms showing the total ROS levels in the cortex (**A**). Error bars represent mean ± SD. (** *p* < 0.01, *** *p* < 0.001 and **** *p*< 0.0001 vs. indicated group). *n* = 5 in each group. MCAO, middle cerebral artery occlusion-reperfusion; GFP, green fluorescent protein; AAV, ATF4 overexpression adeno-associated virus; siRNA, small interfering RNA.

ATF4 has the ability to increase parkin expression and inhibit NLRP3 inflammasome activation, but the role of parkin in the suppression of NLRP3 inflammasome activation is still unknown. As demonstrated in the above results, we successfully used parkin-siRNA to decrease parkin expression. As depicted in Figure 6B–F, the protein levels of NLRP3, cleaved-caspase-1, cleaved-IL-1β and cleaved-IL-18 in the AAV+MCAO group were reduced compared with the MCAO group. Notably, such a reduction was significantly reversed by a subsequent parkin-siRNA treatment. Treatment with parkin-siRNA also reversed the negative effect of ATF4 on the production of ROS (Figure 6A).

Taken together, these findings suggest that parkin is essential for the ATF4-mediated inhibition of the NLRP3 inflammasome activation in cerebral I/R injury.

### 3.6. ATF4 Inhibits NLRP3 Inflammasome Activation through Mitophagy Induction

**Figure 7:**
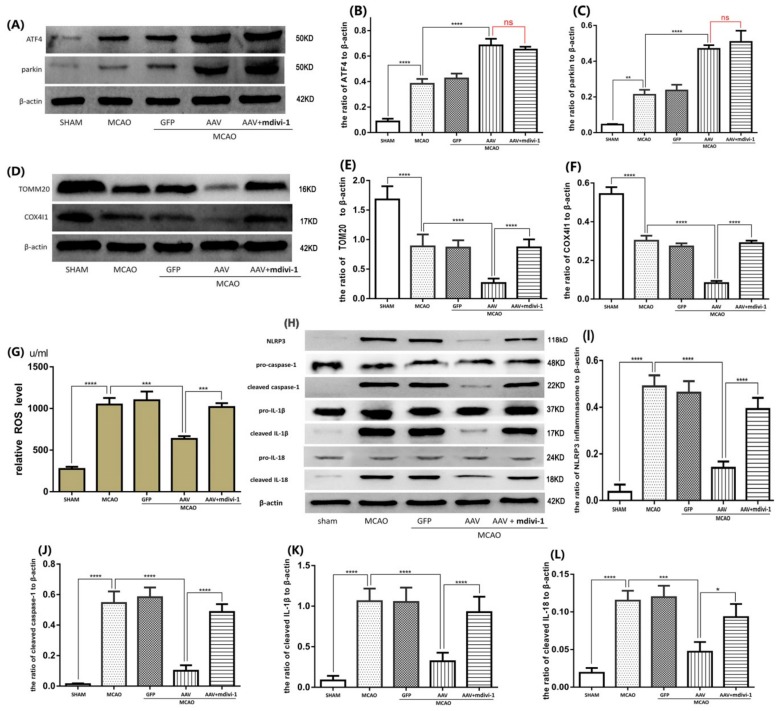
Mdivi-1 treatment impaired the effects of ATF4 on mitophagy activity and NLRP3 inflammasome activation. (**A**–**C**) No differences were observed in terms of ATF4 and parkin expression between the AAV + MCAO group and the mdivi-1 + AAV + MCAO group. Mdivi-1 treatment significantly impaired the inhibitory effects of ATF4 on TOM20 and COX4I1 expressions (**D**–**F**), NLRP3 inflammasome activation (**H**–**L**) and ROS production (**G**). Error bars represent mean ± SD. (* *p* < 0.05, ** *p* < 0.01, *** *p* < 0.001 and **** *p*< 0.0001 vs. indicated group). *n* = 5 in each group. MCAO, middle cerebral artery occlusion-reperfusion; GFP, green fluorescent protein; AAV, ATF4 overexpression adeno-associated virus; siRNA, small interfering RNA; mdivi-1, mitochondrial division inhibitor-1.

It has been reported that mdivi-1 is a specific inhibitor of mitophagy by blocking DNM1L, which is a key regulator of mitochondrial fission and subsequent autophagic elimination [3,28]. To investigate whether mitophagy contributes to the inhibitory effect of ATF4 on NLRP3 inflammasome activation, rats undergoing cerebral I/R stimulation were pretreated with the AAV and then administered with mdivi-1. As expected, AAV treatment reduced TOM20 and COX4I1 expressions compared to the MCAO group. Treatment with mdivi-1 significantly inhibited the AAV-mediated TOM20 and COX4I1 down-regulation (Figure 7D–F). Importantly, no significant changes in the protein levels of ATF4 and parkin were observed between the AAV+MCAO group and the mdivi-1+ AAV+MCAO group (Figure 7A–C). These data confirmed that mdivi-1 administration could be able to block the ATF4-induced augmentation of mitophagy. Additionally, we have also found that the inhibitory effects of ATF4 on the expressions of NLRP3, cleaved-caspase-1, cleaved-IL-1β and cleaved-IL-18 were significantly attenuated by treatment with mdivi-1 administration (Figure 7H–L). Consistent with these results, the blockade of mitophagy by mdivi-1 administration also attenuated the preventive effect of AAV treatment on ROS (Figure 7G).

Collectively, these results demonstrated that parkin-dependent mitophagy activity is required for the inhibitory effect of ATF4 on NLRP3 inflammasome-mediated inflammation during cerebral I/R injury.

## 4. Discussion

Although the concept of NLRP3 inflammasome has been proposed for many years, the contribution and regulatory mechanisms of NLRP3 inflammasome activation in cerebral I/R injury have not been entirely elucidated. In this present experiment, we have demonstrated that ATF4 exerts a protection against cerebral I/R injury and inhibits NLRP3 inflammasome activation. Additionally, we also have proved that ATF4 has the ability to up-regulate parkin expression and augment mitophagy activity during cerebral I/R injury. Notably, treatment with parkin-siRNA not only prevented the ATF4-induced increase of mitophagy activity but also reversed the inhibition of ATF4 on NLRP3 inflammasome-mediated inflammatory response. Moreover, ATF4-mediated inhibition of NRLP3 inflammasome activation were clearly attenuated by mdivi-1 administration. Altogether, such results support the hypothesis that ATF4 may protect against cerebral I/R injury by suppressing NLRP3 inflammasome-mediated inflammation and that parkin-dependent mitophagy activity is required for this neuroprotective effect.

Inflammasome can mediate neurocytes death in ischemic stroke via a number of mechanisms, including the productions of pro-inflammatory factors and the pleiotropic effects of cleaved caspase-1 in mediating apoptosis and pyroptosis [1]. As the first identified inflammasome in cerebral ischemic injury, NLRP1 inflammasome could activate precursor caspase-1 to produce both mature IL-1β and IL-18 to mediate neurocytes death after ischemic stroke [32]. As another important member of inflammasomes, NLRP3 inflammasome, which is receiving more attention and has been better characterized in recent years, is involved in a number of diseases, including type II diabetes mellitus, renal disease and neurodegenerative disorders [33]. In addition, several studies indicated that activated NLRP3 inflammasome and its related cytokines significantly contribute to cerebral I/R injury [15,16,34]. Furthermore, a previous report from our laboratory showed the knockdown of NLRP3 using siRNA alleviated cerebral I/R injury as evidenced by a decreased infraction volume, improved neurological scores and reduced cerebral edema [35]. Another research from our team suggested that the inhibition of NLRP3 inflammasome activation induced by sulforaphane exerts a neuroprotective effect in cerebral I/R injury [36]. Moreover, we also reported that resveratrol-induced autophagy activity protects against cerebral I/R injury by inhibiting NLRP3 inflammasome activation [31]. Therefore, these findings collectively indicate that activated NLRP3 inflammasome exerts a pivotal role in the pathogenesis of cerebral I/R injury and that inhibition of NLRP3 inflammasome activation become a potential strategy for hampering the development of cerebral I/R injury.

ATF4, one of the major ERS (Endoplasmic Reticulum Stress) response effectors, is involved in a variety of diseases and its role in cell survival and death is not completely clear [37]. Upon severe or prolonged ER stress, activation of the PERK-elF2α-ATF4-CHOP signaling cascade is responsible for ER stress-induced apoptotic cell death as reported by several studies [38,39]. In contrast, other studies showed a pro-survival function of ATF4. Sun et al. reported that ATF4 exerted protection against neuronal death in the cellular model of Parkinson disease [25]. Wu and colleagues indicated that ATF4 up-regulation was critical in the salubrinal-mediated protection against rotenone-induced dopaminergic cell death [27]. Kim et al. suggested that ATF4-induced sestrin 2 up-regulation was responsible for the protection of carbon monoxide against hepatic steatosis [40]. However, both the effect of ATF4 on cerebral I/R injury and its underlying mechanism are not fully elucidated. In this work, we have demonstrated that ATF4 overexpression alleviates cerebral I/R injury as demonstrated by a reduced infraction volume, decreased neurological scores and improved outcomes of HE and Nissl staining. Meanwhile, ATF4 overexpression decreased the levels of activated NLRP3 inflammasome, cleaved caspase-1, cleaved IL-1β and cleaved IL-18 as well as mtROS following cerebral I/R injury. Notably, ATF4 knockdown had the opposite effect on the cerebral I/R injury and NLRP3 inflammasome-mediated inflammatory cascade. These above findings suggest that ATF4 may exert a neuroprotective effect in cerebral I/R injury through the inhibition of NLRP3 inflammasome activation. In support of this view, is a study reporting that ATF4 could protect neurons against ER stress-induced cell death by regulating parkin expression in the reperfusion phase after cerebral ischemia [28]. Contrary to our view, recent papers suggest that ATF4 may aggravate ER stress-induced neuronal apoptosis through CHOP activation in transient cerebral ischemic injury [41,42,43]. The reason for such inconsistent results may be due to different ATF4-regulated targets. Indeed, when moderate ER stress occurs, the unfolded protein response (UPR) and ATF4-mediated proteins, such as parkin, can protect the cell; however, in the case of severe ER stress, the elF2α-ATF4-CHOP pathway is activated, ultimately leading to cell apoptosis [44].

Damaged mitochondria and their released mitochondrial signals play a pivotal role in regulating NLRP3 inflammasome activation [45]. Mitophagy is a primary mechanism in controlling mitochondrial quality through the removal of dysfunctional ones. Mitophagy induction has been reported to negatively regulate NLRP3 inflammasome activation, preventing excessive inflammation in LPS-primed BMDM [23]. Thus, we investigated whether mitophagy was involved in the inhibitory effect of ATF4 on NLRP3 inflammasome activation during cerebral I/R injury. Our experiments indicated that ATF4 overexpression was able to enhance mitophagy activity, whereas ATF4-siRNA had the opposite effect. Notably, when mitophagy was blocked by mdivi-1 treatment, the inhibitory effects of ATF4 on NLRP3 inflammasome-mediated inflammation and ROS production were clearly abolished, revealing that mitophagy induction is required for the inhibition of ATF4 in NLRP3 inflammasome activation during cerebral I/R injury. Consistent with our opinion, Guo and colleagues reported that mitophagy induction was responsible for Andro-mediated suppression of NLRP3 inflammasome activation in colitis-associated cancer [46]. Yang et al. reported that defective mitophagy contributed to ROS production and NLRP3 inflammasome-driven inflammatory response under PA stress in type 2 diabete [47]. Kim et al. suggested that sestrin2 inhibited NLRP3 inflammasome activation in macrophages by inducing mitophagy [48]. Altogether, these findings along with our experimental results suggest that ATF4 may suppress NLRP3 inflammasome-mediated inflammation by enhancing mitophagy activity and that mitophagy induction could negatively regulate NLRP3 inflammasome activation through ROS down-regulation.

During the process of mitophagy, parkin is recruited to dysfunctional mitochondria where it interacts with other autophagic proteins, including p62 and LC3, to induce mitophagy [17]. The role of PINK1-Parkin in the mitophagy process has been intensively studied. However, the upstream regulative mechanism of PINK1/Parkin-mitophagy has not been fully described. Interestingly, ATF4 has been reported to transcriptionally upregulate the expression of Parkin [26,27]. Thus, we further explored the role of parkin in the ATF4-induced mitophagy activity and inhibition of NLRP3 inflammasome activation during cerebral I/R injury. In this study, we have confirmed that ATF4 overexpression significantly increased parkin expression, enhanced mitophagy activity and inhibited NLRP3 inflammasome activation in cerebral I/R injury. Intriguingly, these effects induced by ATF4 were effectively abrogated through treatment with parkin-siRNA. These results suggest that ATF4 regulation of mitophagy activity and NLRP3 inflammasome activation depends on parkin expression. In agreement with our study, a previous research reported that the EIF2S1-ATF4-parkin-mitopahgy pathway is required for the protective effect of ER stress induced by TG and TM in cerebral I/R injury [28]. Compared with such a result, our data further highlight the suppressive effects of the ATF4-parkin-mitophagy axis on NLRP3 inflammasome activation, indicating that mitophagy induction may protect against cerebral I/R injury by suppressing NLRP3 inflammasome activation during TG and TM-induced ER stress. In addition, other regulators of mitophagy have been identified, such as FUNDC1 and BNIP3/NIX [24,49]. However, the potential role of such proteins in regulating mitophagy activity during cerebral I/R injury has not been investigated. Thus, more efforts are needed to address this issue.

## 5. Conclusions

In summary, the present study firstly confirmed that ATF4 may alleviate cerebral I/R injury by negatively regulating NLRP3 inflammasome-induced inflammation cascade and that the increase of parkin-dependent mitophagy activity is responsible for the inhibition of ATF4 in NLRP3 inflammasome activation. This conclusion may provide a new strategy to relieve cerebral I/R injury by modulating mitophagy-NLRP3 inflammasome axis.

## Supplementary Materials

The following are available online at https://www.mdpi.com/2073-4409/8/8/897/s1, Table S1: The siRNAs suquence for ATF4 and Parkin. Table S2: The primeirs sequence for ATF4 and β-actin. Figure S1: The expressions of ATF4 and NLRP3 inflammasome at different reperfusion time points after MCAO. Figure S2: The knockdown efficiency of three different siRNAs on ATF4 expression. Figure S3: The knockdown efficiency of three different siRNAs on parkin expression.

## Abbreviations

I/R	Ischemia/reperfusion
MCAO	middle cerebral artery occlusion-reperfusion
siRNA	small interfering RNA
AAV	adeno-associated virus
GFP	Green fluorescent protein
NC	negative control siRNA
BSA	bovine serum albumin
ATF4	Activating Transcription Factor 4
COX4I1	cytochrome c oxidase IV isoform 1
TOM20	translocase of outer mitochondrial membrane 20
ROS	reactive oxygen species
NLRP3	Nod-like receptor protein 3
Mdivi-1	mitochondrial division inhibitor-1
