# Supplementary material for: Parkin-Dependent Mitophagy Is Required for the Inhibition of ATF4 on NLRP3 Inflammasome Activation in Cerebral Ischemia-Reperfusion Injury in Rats
Source: Cells. 2019 Aug 14;8(8):897. doi: 10.3390/cells8080897 (PMC6721752; doi:10.3390/cells8080897)
Supplement: Supplementary file 1 [file cells-08-00897-s001.pdf]

Table 1. Information of siRNA on ATF4 and pakin.

Three different siRNAs of ATF4 or parkin were designed and chemically synthesized. The sequences of these different siRNAs were represented in table 1. (NC siRNA: Stable Negative Control siRNA).

Gene ID	Gene Name		sense (5'—3')	antisense (5'—3')
79255	ATF4	NC siRNA	UUCUCCGAACGUGUCACGUTT	ACGUGACACGUUCGGAGAATT
		951 siRNA	GCGAGUGUAAAGAGCUAGATT	UCUAGCUCUUUACACUCGCTT
		135 siRNA	GUCUCUUAGAUGACUAUCUTT	AGAUAGUCAUCUAAGAGACTT
		626 siRNA	GCUGCUUAUAUUACUCUAATT	UUAGAGUAAUAUAAGCAGCTT
56816	parkin	NC siRNA	UUCUCCGAACGUGUCACGUTT	ACGUGACACGUUCGGAGAATT
		883 siRNA	GCUCAACGAUCGGCAGUUUTT	AAACUGCCGAUCGUUGAGCTT
		949 siRNA	CCAACUCCCUGAUUAAAGATT	UCUUUAAUCAGGGAGUUGGTT
		253 siRNA	GGAACAACAGAGUAUCGUUTT	AACGAUACUCUGUUGUUCCTT

Table 2. Information on ATF4 andβ-actin primer.

The primer sequences of ATF4 and β-actin used in qPCR were represented in table 2.

Gene symbol	GenBank accession no.	Primer sequences	Product size (b.p.)
ATF4	NM_024403.2	Forward: CCGAGATGAGCTTCCTGAAC	121
		Reverse: TTGGCCACCTCCAGATAGTC	121
β-actin	NM_031144.3	Forward: CACCCGCGAGTACAACCTTC	207
		Reverse: CCCATACCCACCATCACACC	207

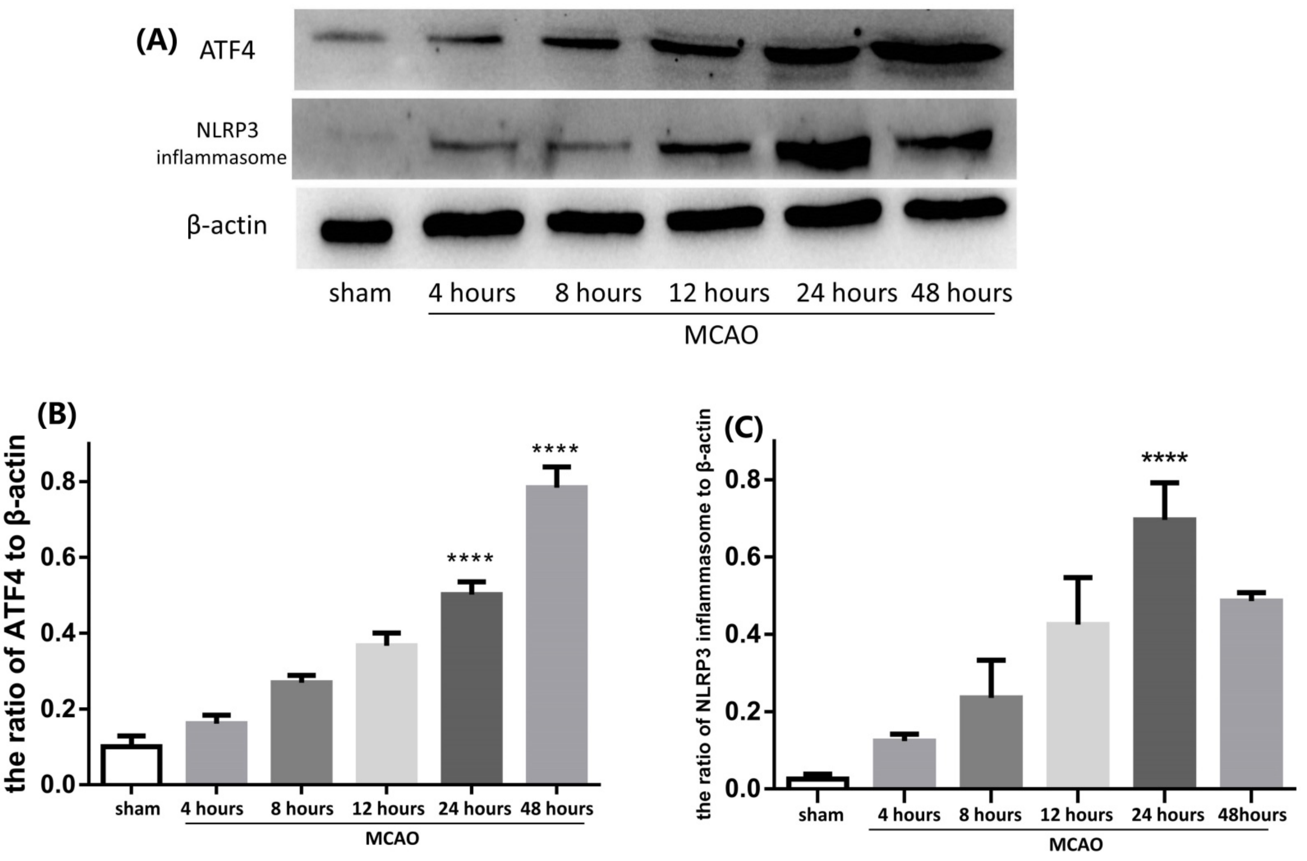

Figure S1: The expressions of ATF4 and NLRP3 inflammasome at different reperfusion time points after MCAO.

(A, B) Compared with the sham group, ATF4 expression increased remarkably at 24 hours after reperfusion, then, continuously increased at 48 hours reperfusion. (A, C) Compared with the sham group, the expression of NLRP3 inflammasome increased gradually and peaked at 24 hours after reperfusion, however, decreased at 48 hours reperfusion. Error bars represent mean ± SD. (****$p < 0.0001$ vs the sham group). Experiment was repeated at least three times.

Therefore, 24 hours reperfusion after MCAO was chosen as the specific time point for our experiment.

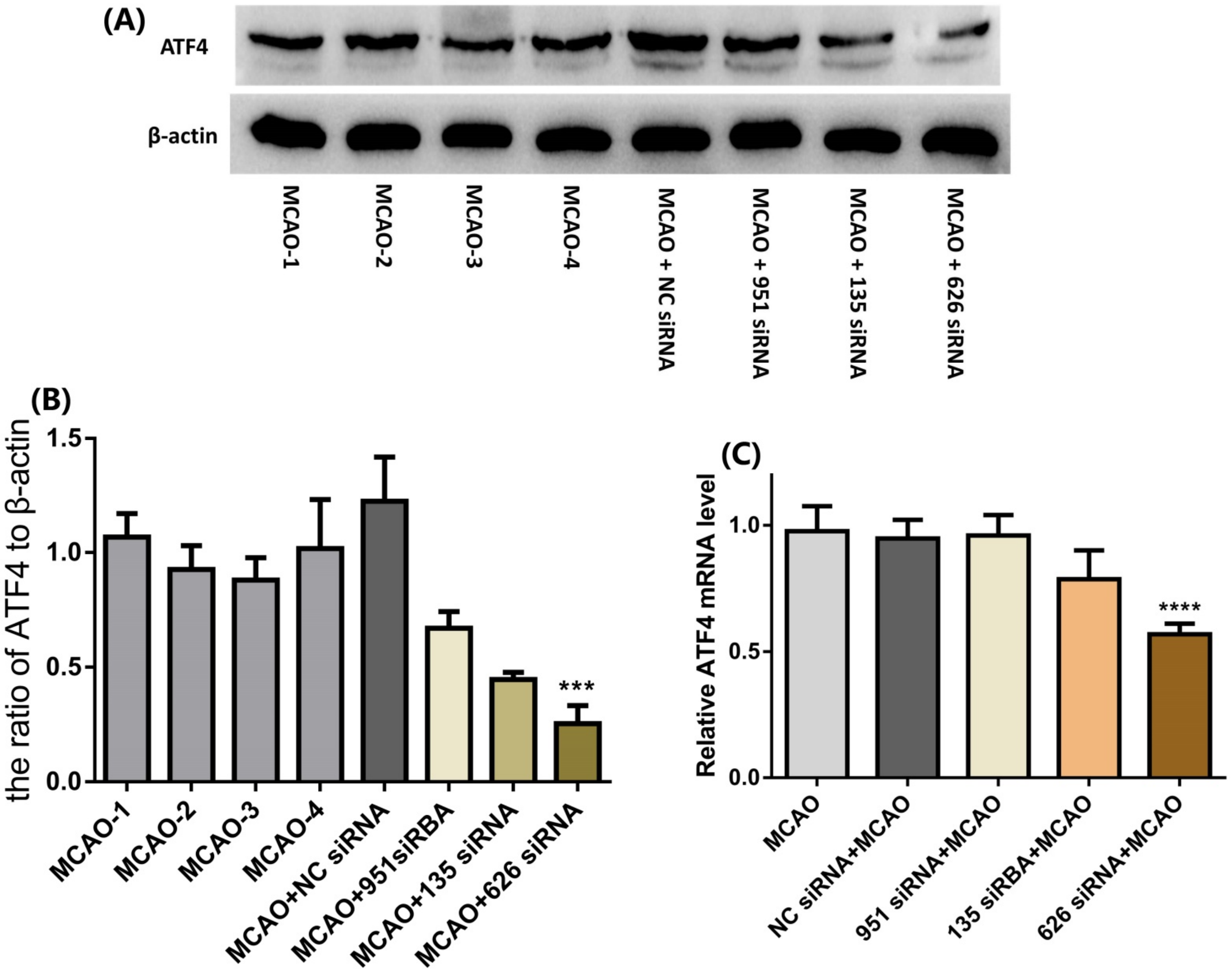

Figure S2: The knockdown efficiency of three different siRNAs on ATF4 expression.

(A, B) The protein level of ATF4 was decreased most significantly by treatment with 626 siRNA as compared with the MCAO groups. (C) The mRNA level of ATF4 was reduced most obviously by 626 siRNA treatment when compared to the MCAO group. Error bars represent mean ± SD. (***p< 0.001 vs the MCAO groups). Experiment was repeated at least three times.

Take together, 626 siRNA was the most effective in ATF4 knockdown. Thus, 626 siRNA was used for ATF4 knockdown in our experiment.

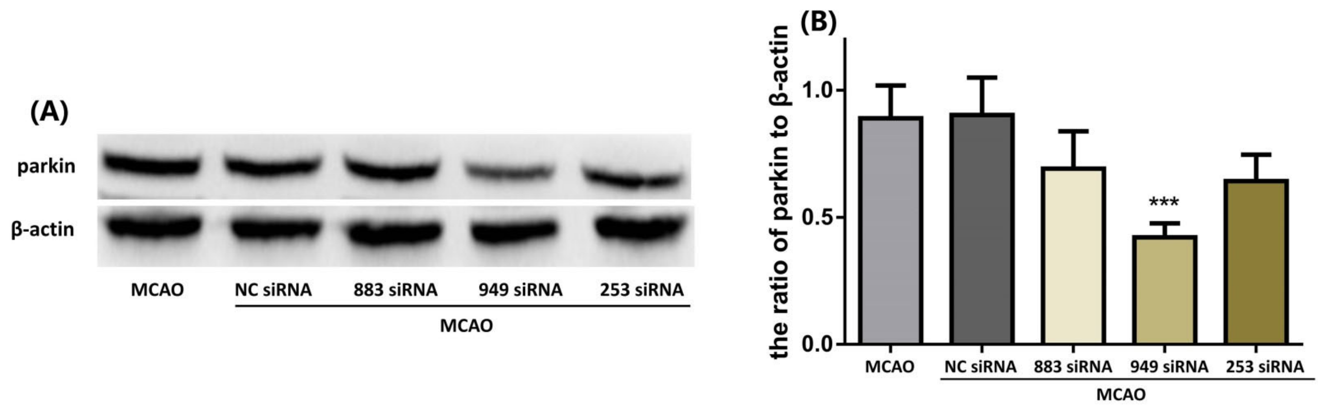

Figure S3: The knockdown efficiency of three different siRNAs on parkin expression.

(A, B) The parkin expression was decreased most remarkably by 949 siRNA treatment compared to the MCAO group. Error bars represent mean ± SD. (***$p < 0.001$ vs the MCAO group). Experiment was repeated at least three times.

Therefore, 949 siRNA was used to knockdown the expression of parkin in our experiment.

The details of ELISA procotol.

1. Reagent Preparation

1. Standard Solutions

- 10,000 pg/mL: Add 1 mL of sample diluent buffer into one tube of standard (10 ng per tube) and mix thoroughly. Note: Store this solution at 4°C for up to 12 hours (or -20°C for 48 hours) and avoid freeze-thaw cycles.
- 5,000 pg/mL: Mix 0.3 mL of 10,000 pg/mL with 0.3 mL of sample diluent buffer and mix thoroughly.
- 2,500 pg/mL: Mix 0.3 mL of 5,000 pg/mL with 0.3 mL of sample diluent buffer and mix thoroughly.
- Perform similar dilutions until the standard solutions with these concentrations (pg/mL) are made:
- 1,250, 625, 312, 156 and 78.
- Add 100 μL of each of the diluted standard solutions to the appropriate empty wells. Repeat in duplicate or triplicate for accuracy.

Note: The standard solutions are best used within 2 hours.

2. Biotinylated Antibody

- Calculate the total volume needed for the assay by multiplying 0.1 mL/well and the number of wells required. Add 2-3 extra wells to the calculated number of wells to account for possible pipetting errors.
- Generate the required volume of diluted antibody by performing a 1:100 dilution (For each 1 μL concentrated antibody, add 99 μL antibody dilution buffer) and mixing thoroughly.

3. Avidin-Biotin-Peroxidase Complex (ABC)

- Calculate the total volume needed for the assay by multiplying 0.1 mL/well and the number of wells required. Add 2-3 extra wells to the calculated number of wells to account for possible pipetting errors.
- Generate the required volume of diluted ABC solution by performing a 1:100 dilution (For each 1 μL concentrated ABC solution, add 99 μL ABC dilution buffer) and mixing thoroughly.

Note: The diluted ABC solution should not be prepared more than 1 hour prior to the experiment.

2. Sandwich ELISA Protocol

All of the ELISA kits from Boster use the sandwich format and biotin-streptavidin chemistry. Our ELISA assays require the dilutions of standard solutions, biotinylated antibody (detection antibody) and avidin-biotin-peroxidase complex.

1. Capture Antibody Coating

(These steps are not required if the pre-adsorbed Picokine ELISA kits from Boster are used)

- Dilute the capture antibody to a final concentration of 1-10 μg/mL in bicarbonate/carbonate antigen-coating buffer (100 mM NaHCO3 in deionized water; pH adjusted to 9.6).
- Pipette 100 μL of diluted antibody to each well of a microtiter plate.
- Cover the plate with adhesive plastic and incubate at 4°C overnight (or 37°C for 30 min).
- Remove the coating solution and wash the plate 3X with 200 μL PBS (Phosphate Buffered Saline) buffer (10 mM Na2HPO4 and 1.8 mM NaH2PO4 in deionized water with 0.2% Tween 20; pH Adjusted to 7.4) with for 5 minutes each time. The coating/washing solutions can be removed by flicking the plate over a sink. The remaining drops can be removed by patting the plate on a paper towel or by aspiration. Do not allow the wells to dry out at any time.

2. Blocking

(These steps are not required if the pre-adsorbed Picokine ELISA kits from Boster are used)

- Pipette 200 μL blocking buffer (5% w/v non-fat dry milk in PBS buffer) per well to block residual protein-binding sites. Alternatively, BSA or BlockACE can be used to replace non-fat dry milk.
- Cover the plate with adhesive plastic and incubate for 1-2 hour(s) at 37°C (or at 4°C overnight).
- Remove the blocking solution and wash the plate 2X with 200 μL PBS for 5 minutes each time. Flick the plate and pat the plate as described in the coating step.

3. Reagent Preparation

- Prepare for the diluted standard solutions, biotinylated antibody and ABC solutions as shown in the above Reagent Preparation section.

Note: The diluted ABC solution should not be prepared more than 1 hour prior to the experiment.

4. Sample (Antigen) Incubation

- Serially dilute the sample with blocking buffer immediately before use. The optimal dilution should be determined by a titration assay according to the antibody manufacturer.
- Pipette 100 μL of each of the diluted sample solutions and control to each empty well. Repeat in duplicate or triplicate for accuracy. The negative control should be species- and isotype-matched as well as non-specific immunoglobulin diluted in PBS buffer.
- Cover the plate with adhesive plastic and incubate for 2 hours at room temperature.
- Remove the content in the wells and wash them 3X with 200 μL PBS buffer for 5 minutes each time. Flick the plate and pat the plate as described in the coating step.

5. Biotinylated Antibody Incubation

- Pipette 100 µL of diluted antibody to the wells with control, standard solutions and diluted samples.
- Cover the plate with adhesive plastic and incubate for 1 hour at 37°C (or 2 hours at room temperature). These incubation times should be sufficient to receive a strong signal. However, if a weak signal is observed, perform incubation overnight at 4°C for a stronger signal.
- Remove the content in the wells and wash them 3X with 200 µL PBS for 5 min each time. Flick the plate and pat the plate as described in the coating step.

6. ABC Incubation

- Pipette 100 µL of diluted ABC solution to the wells with control, standard solutions and diluted samples.
- Cover the plate with adhesive plastic and incubate for 0.5 hour at 37°C.
- Remove the content in the wells and wash them 3X with 200 µL PBS buffer for 5 min each time. Flick the plate and pat the plate as described in the coating step.

7. Substrate Preparation

Prepare the substrate solution immediately before use or bring the pre-made substrate to room temperature. The two widely used enzymes for signal detection are horse radish peroxidase (HRP) and alkaline phosphatase (AP), and their corresponding substrates, stopping solutions, detection absorbance wavelengths and color developed are as follows:

Enzyme	Substrate*	Stop Solution	Absorbance (nm)	Color Developed
HRP	TMB	2M H2SO4	450	Yellow
AP	pNPP	0.75M NaOH	405	Yellow

* TMB: 3,3’,5,5’-tetramethylbenzidine; pNPP: p-nitrophenyl-phosphate

Note:

- The TMB substrate must be kept at 37°C for 30 min before use.
- Hydrogen peroxide can also act as a substrate for HRP.
- Sodium azide is an inhibitor of HRP. Do not include the azide in buffers or wash solutions if HRP-labeled conjugate is used for detection.

8. Signal Detection

- Pipette 90 µL of substrate solution to the wells with the control, standard solutions and diluted samples.
- Incubate the plate at 37°C in the dark. If TMB is used, shades of blue will be observed in the wells with the most concentrated solutions. Other wells may show no obvious color.
- Color should be developed in positive wells after 15 min. After sufficient color development, pipette 100 µL of stop solution to the appropriate wells (if necessary).

- Read the absorbance (OD: Optical Density) of each well with a plate reader.

9. Data Analysis

- Prepare a standard curve using the data produced from the diluted standard solutions. Use absorbance on the Y-axis (linear) and concentration on the X-axis (log scale).
- Interpret the sample concentration from the standard curve.
